# Low‐dose digoxin improves cardiac function in patients with heart failure, preserved ejection fraction and atrial fibrillation – the RATE‐AF randomized trial

**DOI:** 10.1002/ejhf.70022

**Published:** 2025-09-02

**Authors:** Karina V. Bunting, Asgher Champsi, Simrat K. Gill, Khalil Saadeh, A. John Camm, Mary Stanbury, Sandra Haynes, Jonathon N. Townend, Richard P. Steeds, Dipak Kotecha, the RAte control Therapy Evaluation in Permanent Atrial Fibrillation (RATE‐AF) Trial Group

**Affiliations:** ^1^ Institute of Cardiovascular Science University of Birmingham Birmingham UK; ^2^ University Hospitals Birmingham NHS Foundation Trust Birmingham UK; ^3^ NIHR Birmingham Biomedical Research Centre Birmingham UK; ^4^ NHS West Midlands Secure Data Environment University Hospitals Birmingham NHS Foundation Trust Birmingham UK; ^5^ Cardiology Clinical Academic Group Molecular & Clinical Sciences Institute, City St George's University of London London UK; ^6^ Patient and Public Involvement team Birmingham UK

**Keywords:** Digoxin, Beta‐blockers, Heart failure with preserved ejection fraction, Atrial fibrillation, Randomized controlled trial, Echocardiography

## Abstract

**Aims:**

To compare the effect of digoxin versus beta‐blockers on left ventricular function, in patients with permanent atrial fibrillation (AF) and symptoms of heart failure within the RATE‐AF randomized trial.

**Methods and results:**

Blinded echocardiograms were performed at baseline and 12‐month follow‐up using a pre‐defined imaging protocol and the index‐beat approach. The change in systolic and diastolic function was assessed, stratified by left ventricular ejection fraction (LVEF). Overall, 145 patients completed follow‐up, with median age 75 years (interquartile range 69–82) and 44% women. In 119 patients with baseline LVEF ≥50%, a significantly greater improvement in systolic function was noted in patients randomized to low‐dose digoxin versus beta‐blockers: adjusted mean difference for LVEF 2.3% (95% confidence interval [CI] 0.3–4.2; *p* = 0.021), s′ 1.1 cm/s (95% CI 1.0–1.2; *p* = 0.001) and stroke volume 6.5 ml (95% CI 0.4–12.6; *p* = 0.037), with no difference in global longitudinal strain (*p* = 0.11) or any diastolic parameters. There were no significant differences between groups for patients with LVEF 40–49% and <40%. Digoxin reduced N‐terminal pro‐B‐type natriuretic peptide compared to beta‐blockers (geometric mean difference 0.77; 95% CI 0.64–0.92; *p* = 0.004), improved New York Heart Association functional class (odds ratio [OR] 11.3, 95% CI 4.3–29.8; *p* < 0.001) and modified European Heart Rhythm Association arrhythmia symptom class (OR 4.91, 95% CI 2.36–10.23; *p* < 0.001), with substantially less adverse events (incident rate ratio 0.21, 95% CI 0.13–0.31; *p* < 0.001). There were no interactions between treatment effects and baseline LVEF for these outcomes (interaction *p* = 0.62, 0.49, 0.07 and 0.13, respectively).

**Conclusions:**

Low‐dose digoxin in patients with symptoms of heart failure, preserved LVEF and permanent AF leads to a significantly greater improvement in systolic function compared to treatment with beta‐blockers.

## Introduction

Atrial fibrillation (AF) is the commonest heart rhythm condition and is predicted to double further in prevalence over the next 20 years.^1^ Half of patients with AF also suffer from heart failure, which worsens prognosis, increases the risk of hospitalization and places increased strain on hospital services.^2^ Heart failure with preserved ejection fraction (HFpEF) is the predominant type of heart failure in AF, with limited treatment options available.^3^, ^4^

The choice of rate control agent in patients with AF and heart failure has not previously been evidence‐based, and often relies on clinician preference or prior experience.^5^ The RAte control Therapy Evaluation in permanent Atrial Fibrillation (RATE‐AF) trial was the first head to head, randomized controlled trial (RCT) of beta‐blockers versus digoxin, the two most commonly used medications in this patient group.^6^ RATE‐AF demonstrated that low‐dose digoxin had the same impact on physical‐related quality of life as beta‐blockers, but with substantially better functional improvement, lower natriuretic peptide release and significantly fewer adverse events. Previous studies have found that rate control can improve ventricular function in patients with AF,^7^, ^8^ however it is unknown whether different rate control therapies have a distinct impact on either systolic or diastolic cardiac performance. This study provides a direct randomized comparison of low‐dose digoxin with beta‐blocker therapy on blinded echocardiography data, with a focus on patients with AF and a preserved ejection fraction where there are limited data to support clinical decision‐making.

## Methods

### Patient population

RATE‐AF was a prospective, randomized, open‐label, blinded endpoint trial comparing heart rate control using digoxin versus beta‐blockers. Approval was obtained from the East Midlands Derby Research Ethics Committee (16/EM/0178), the Health Research Authority (IRAS 191437), and the Medicines and Healthcare Products Regulatory Agency. The trial was funded by the UK National Institute for Health and Care Research (NIHR; CDF‐2015‐08‐074). Trial registration: ClinicalTrials.gov Identifier: NCT02391337 and clinicaltrialsregister.eu Identifier: 2015‐005043‐13.

The rationale, design and main results from the trial have previously been published, along with the trial protocol.^6^, ^9^ The trial was co‐designed and co‐run with a patient and public involvement team.^10^ The trial was embedded into usual care within the National Health Service (NHS), with patients recruited from primary care practices and three hospitals in the West Midlands region of England from 2016 to 2018. All participants provided written informed consent after review of patient information developed by the patient and public involvement team. The inclusion criteria were age 60 years or older, permanent AF only (characterized at time of randomization, as a physician decision for rate control with no plans for cardioversion, anti‐arrhythmic medication, or ablation therapy), and symptoms suggestive of heart failure with New York Heart Association (NYHA) class II or above. Key exclusion criteria were heart rate <60 bpm or evidence of prior second or third‐degree heart block, with few other exclusion criteria to allow results to be applicable to a wide population (see online supplementary *Table Appendix* *S1* for the full list of selection criteria). There were no exclusion criteria for echocardiographic image quality or heart failure according to left ventricular ejection fraction (LVEF), except for patients with decompensated heart failure in the last 14 days evidenced by the need for intravenous inotropes, vasodilators or diuretics.

### Randomization

Randomization was determined using a computer‐generated minimization algorithm, stratified by the modified European Heart Rhythm Association (mEHRA) class and gender. The treatment allocation was concealed until all baseline assessments were completed. Participants were randomized to low‐dose digoxin (62.5–250 μg daily) or bisoprolol (1.25–10 mg daily) on a 1:1 ratio. Those with intolerance to bisoprolol were permitted to receive another beta‐blocker at equivalent dose. There was no cross‐over between randomized groups.

### Echocardiography

Transthoracic echocardiography was performed at baseline and 12‐month follow‐up using a Philips EPIQ 7 and X5‐1 transducer, performed by the same British Society of Echocardiography level 2 accredited sonographer with over 5 years of experience. The sonographer was blinded to the patient's clinical details and trial therapy. All echocardiograms were conducted following a pre‐specified protocol ratified before the trial started (online supplementary *Table* *S2*). A minimum of 30‐beat loops or 30‐beat Doppler traces were obtained for all measurements of systolic and diastolic function. For loops of the apical 2‐, 3‐ and 4‐chamber views depth, sector width and focus were optimized to maximize frame rate and ensure all left ventricular endocardial segments were well visualized. The left atrium was assessed in both the apical 2‐ and 4‐chamber views. Doppler traces were obtained on tissue Doppler imaging (TDI) derived from the lateral and septal wall, mitral inflow, pulmonary venous flow and left ventricular outflow tract flow.

No measurements were taken at the time of imaging; all measurements were calculated offline at least 3 months after the patient's echocardiogram by the same echocardiographer. To ensure blinding at the time of analysis, each echocardiogram study was given a unique alphanumeric code, and all patient identifiable features (including trial number) were removed. Analysis was carried out using Philips Q‐station (version 3.5; Philips Healthcare, Andover, MA, USA). All measurements were calculated as the average of three index‐beats (online supplementary *Figure Appendix* *S1*), defined as the cardiac cycle following two cycles of similar duration (within 60 ms of each other).^11^ Systolic measurements assessed were: LVEF using the modified Simpson's biplane approach, global longitudinal strain (GLS), TDI myocardial systolic velocity (s′) from the lateral and septal walls, and stroke volume derived from the continuity equation. Diastolic measurements assessed were: mitral inflow velocity (E) and E‐wave deceleration time, TDI‐derived myocardial early diastolic velocity (e′), the ratio of mitral E to average of e′ (E/e′), isovolumic relaxation time, pulmonary venous diastolic deceleration time, tricuspid regurgitation maximal velocity (TR V_max_), left atrial reservoir strain and left atrial ejection fraction.

### Clinical outcomes

Potential interactions of randomized therapy with LVEF were assessed using secondary outcomes obtained in the RATE‐AF trial. N‐terminal pro‐B‐type natriuretic peptide (NT‐proBNP) was measured on the same day as the baseline and 12‐month echocardiograms, with the clinical and imaging team blinded to results. Symptoms and functional impact were assessed using the NYHA and mEHRA classification systems for heart failure and AF, respectively, determined by the clinical team who were blinded to the results of the echocardiogram. Adverse events were collected at each visit by the research nurse according to the summary of product characteristics for each drug. All serious adverse events and incident cardiovascular events underwent a process of independent clinical adjudication.

### Statistical analysis

Summary results are presented as number (percentage), mean with standard deviation (SD), or median with interquartile range (IQR; displayed as 25th–75th quartiles). The analysis was performed using an intention‐to‐treat analysis (randomized treatment allocation to beta‐blockers or digoxin). Patients were stratified into groups based on baseline LVEF that forms the diagnostic criteria for HFpEF: LVEF ≥50%; heart failure with mildly reduced ejection fraction (HFmrEF): LVEF 41–49%; and heart failure with reduced ejection fraction (HFrEF): LVEF ≤40%. A paired *t*‐test was used to determine the change in echocardiographic parameters from baseline to 12‐month follow‐up, with transformation in cases of non‐normal distribution. Multivariable linear regression models were performed to determine whether there was a difference between treatment groups for the change in systolic and diastolic parameters, stratified by heart failure sub‐type and adjusting for baseline measurement of that echo variable, variables used in the randomization process (mEHRA and gender), age at baseline and history of myocardial infarction. The beta‐blocker arm was used as the reference category for all analysis. Interactions of LVEF with treatment allocation for outcomes (NT‐proBNP, 2 class mEHRA improvement and NYHA class) were evaluated in the fully adjusted models, with the incidence of adverse events during follow‐up assessed using adjusted Poisson regression. Statistical analysis was performed using STATA version 17.0 (StataCorp, College Station, TX, USA). All hypothesis testing was two‐sided, and a two‐tailed *p*‐value of 0.05 was considered statistically significant.

## Results

A plain English summary of results is presented in online supplementary *Table* *S3*. Overall, 160 patients were randomized, completed their baseline visit and were initiated on their allocated treatment. The mean achieved dose in the digoxin group was 161 μg/day (SD 55 μg/day), with mean digoxin level 0.78 ng/ml (SD 0.31 ng/mL). In the beta‐blocker group, the mean dose was 3.2 mg/day of bisoprolol (SD 1.8 mg/day) with alternative beta‐blockers used in seven patients who did not tolerate bisoprolol. A total of 145 participants reached 12‐month follow‐up; 11 died (4 randomized to digoxin, 7 randomized to beta‐blockers) and 3 withdrew and 1 did not attend follow‐up (3 randomized to digoxin, 1 randomized to beta‐blockers) (see online supplementary *Figure* *S2* for consort diagram).

Baseline demographics for the 145 patients in this analysis are shown in *Table* *1*. The median age was 75 years (IQR 69–82), 44% were women, mean NYHA class was 2.4 (SD 0.6), LVEF was 57% (SD 9%) and 52% had clinical signs of heart failure at enrolment. Randomized groups were well balanced apart from a higher proportion with a formal diagnosis of heart failure in the digoxin group, but similar NT‐proBNP and LVEF versus beta‐blockers. Echocardiogram parameters are shown in *Table* *2*; at baseline 119 (82%) patients had preserved LVEF (≥50%), 15 (10%) had mildly reduced LVEF (41%–49%) and 11 (8%) had reduced LVEF (≤40%).

**Table 1:** Characteristics of participants with baseline and 12‐month cardiac imaging

Baseline characteristics	All patients (*n* = 145)	Randomized to digoxin (*n* = 73)	Randomized to beta‐ blockers (*n* = 72)
Age, years, median (IQR)	75 (69–82)	74 (68–79)	79 (70–84)
Female sex, *n* (%)	64 (44)	33 (45)	31 (43)
Body surface area, m^2^, median (IQR)	2.0 (1.8–2.2)	2.1 (1.8–2.2)	2.0 (1.7–2.2)
Body mass index, kg/m^2^, median (IQR)	30.0 (26.3–34.3)	30.3 (26.7–34.7)	29.8 (25.3–33.4)
Treatment for hypertension, *n* (%)	102 (70)	49 (67)	53 (74)
Previous stroke or TIA, *n* (%)	16 (11)	6 (8)	10 (14)
Previous myocardial infarction, *n* (%)	11 (8)	5 (7)	6 (8)
Unplanned admission for AF or HF in the last 12 months, *n* (%)	26 (18)	14 (19)	12 (17)
Previous diagnosis of HF, *n* (%)	51 (35)	32 (44)	19 (26)
Signs of HF on examination, *n* (%)	75 (52)	43 (59)	32 (44)
NYHA class, *n* (%)			
I: no symptoms, no limitations	0 (0)	0 (0)	0 (0)
II: mild symptoms, slight limitation	93 (64)	43 (59)	50 (69)
III: marked symptoms and limitation	47 (32)	28 (38)	19 (26)
IV: symptoms at rest, severe limitation	5 (3)	2 (3)	3 (4)
Mean (SD)	2.4 (0.6)	2.4 (0.6)	2.3 (0.6)
mEHRA class, *n* (%)			
1: no symptoms	0 (0)	0 (0)	0 (0)
2a: mild symptoms not troubling patient	6 (4)	3 (4)	3 (4)
2b: moderate symptoms troubling patient, activity unaffected	70 (48)	32 (44)	38 (53)
3: severe symptoms, activity affected	55 (38)	33 (45)	22 (31)
4: disabling symptoms, activity discontinued	14 (10)	5 (7)	9 (13)
Creatinine, μmol/L, median (IQR)	85 (72–100)	85 (71–95)	86 (75–102)
NT‐proBNP, pg/ml, median (IQR)	1049 (755–1463)	1091 (710–1522)	1011 (753–1404)
Heart rate, bpm, median (IQR)	96 (86–112)	97 (88–113)	95 (85–110)
Systolic/diastolic blood pressure, mmHg, median (IQR)	135/85 (124/77–146/91)	134/85 (124/77–145/91)	136/84 (124/75–148/92)

AF, atrial fibrillation; HF, heart failure; IQR, interquartile range; mEHRA, modified European Heart Rhythm Association; NT‐proBNP, N‐terminal pro‐B‐type natriuretic peptide; NYHA, New York Heart Association; SD, standard deviation; TIA, transient ischaemic attack.

**Table 2:** Echocardiography parameters at baseline and 12 months by treatment group

Parameter	Beta‐blockers (*n* = 72)		Digoxin (*n* = 73)	
	Baseline	12 months	Baseline	12 months
Left ventricular systolic function^a^				
Left ventricular ejection fraction, %, median (IQR)	60.1 (55.1–64.1)	61.4 (56.5–64.6)	57.2 (50.4–62.7)	61.3 (57.5–65.1)
Global longitudinal strain, %, median (IQR)	−14.2 (−12.2 to −15.5)	−15.8 (−14.2 to −17.4)	−13.6 (−11.9 to −14.7)	−15.6 (−13.5 to −17.5)
Average TDI s′, cm/s, median (IQR)	6.7 (5.7–7.6)	6.1 (5.4–7.1)	6.4 (5.7–7.6)	7.0 (6.0–7.9)
Stroke volume, ml, median (IQR)	54 (45–63)	58 (48–69)	58 (48–69)	61 (54–79)
Left ventricular diastolic function^a^				
Average e′, cm/s, median (IQR)	9.7 (8.5–11.1)	8.7 (7.7–10.7)	9.1 (8.1–10.9)	10.1 (7.8–11.3)
Mitral E velocity, cm/s, median (IQR)	93 (79–102)	89 (75–99)	88 (77–101)	92 (79–105)
Mitral deceleration time, ms, median (IQR)	208 (181–235)	224 (201–246)	213 (192–233)	224 (205–249)
Average E/e′, median (IQR)	9.4 (7.5–11.0)	10.0 (8.0–11.8)	9.3 (8.1–11.8)	9.0 (7.5–12.3)
Isovolumic relaxation time, ms, median (IQR)	96 (87–107)	99 (90–110)	97 (89–109)	103 (93–111)
Pulmonary vein ratio, mean (SD)	0.7 (0.6–0.8)	0.6 (0.6–0.7)	0.7 (0.6–0.8)	0.6 (0.6–0.8)
Pulmonary vein deceleration time, ms, median (IQR)	243 (220–257)	257 (235–281)	240 (224–259)	264 (243–285)
Left atrial volume indexed to BSA, ml/m^2^, median (IQR)	44 (36–51)	43 (34–52)	36 (30–46)	39 (34–50)
Left atrial ejection fraction, %, median (IQR)	21.5 (14.7–30.1)	26.3 (18.5–34.0)	23.5 (15.6–34.7)	31.1 (21.9–37.3)
Left atrial reservoir strain, %, mean (SD)	9.7 (3.6)	11.9 (5.5)	10.1 (4.7)	11.8 (4.8)
TR V_max_, m/s, mean (SD)	2.4 (0.4)	2.4 (0.4)	2.3 (0.5)	2.4 (0.5)
Elevated left ventricular filling pressures, *n* (%)^b^	18 (25)	13 (18)	28 (38)	23 (32)
Other parameters of structure and function				
Left ventricular end‐diastolic volume, ml, median (IQR)	76 (56–96)	73 (60–99)	84 (62–106)	87 (69–106)
Left ventricular end‐systolic volume, ml, median (IQR)	29 (22–40)	30 (23–37)	34 (25–44)	33 (25–44)
Moderate/severe valve stenosis, *n* (%)	1 (1)	2 (3)	4 (5)	4 (5)
Moderate/severe valve regurgitation, *n* (%)	10 (14)	10 (14)	15 (21)	9 (12)
Intermediate or high risk of pulmonary hypertension, *n* (%)	13 (18)	22 (31)	11 (15)	24 (33)

BSA, body surface area; E, early diastolic velocity through mitral valve; e′, diastolic tissue velocity; IQR, interquartile range; SD, standard deviation; TDI, tissue Doppler imaging; TR, tricuspid regurgitation.

^a^ Left ventricular systolic and diastolic function were measured on an average of three index‐beats.

^b^ Assessed using the British Society of Echocardiography's diastolic assessment.

### Change in left ventricular function in atrial fibrillation patients with preserved ejection fraction (≥50%)

**Figure 1:**
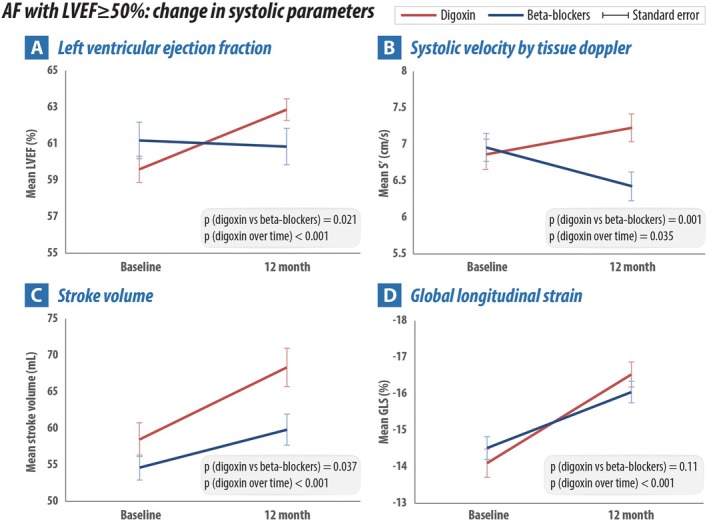
Change in left ventricular systolic parameters from baseline to 12‐month follow‐up in patients with left ventricular ejection fraction (LVEF) ≥ 50% randomized to beta‐blockers versus digoxin. AF, atrial fibrillation; GLS, global longitudinal strain; s′, systolic tissue Doppler velocity.

In patients with LVEF ≥50% at baseline, there was a significant and consistent improvement in systolic function parameters for patients randomized to digoxin (*Table* *3* and *Figure* *1*): from baseline to 12 months an increase in LVEF of 3.3% (*p* < 0.001), change in GLS of −2.4% (*p* < 0.001), increase in TDI s′ by 0.4 cm/s (*p* = 0.035) and increased stroke volume of 9.9 ml (*p* < 0.001). Patients randomized to beta‐blockers had varied results: no significant change in LVEF (baseline to 12 months: −0.3%; *p* = 0.68), an improvement in GLS (−1.5%; *p* < 0.001), worsening of TDI s′ (−0.5 cm/s; *p* = 0.011), but an increase in stroke volume (5.2 ml; *p* = 0.011). In direct comparison by intention‐to‐treat, there was a significantly greater improvement in systolic function parameters in those randomized to digoxin. Adjusted mean differences compared to the beta‐blocker group were 2.3% for LVEF (95% confidence interval [CI] 0.35–4.15; *p* = 0.021), 6.51 ml for stroke volume (95% CI 0.39–12.62; *p* = 0.037) and 1.12 cm/s for TDI‐derived s′ (95% CI 1.05–1.20, *p* = 0.001) (*Table* *3* and *Figure* *1*).

**Table 3:** Change in left ventricular systolic function for digoxin versus beta‐blockers with baseline left ventricular ejection fraction ≥50%

Outcome	Digoxin		Beta‐blockers		Digoxin vs. beta‐blockers
	*n*	Mean change from baseline to 12 months, *p*‐value	*n*	Mean change from baseline to 12 months, *p*‐value	Adjusted mean difference^a^ (95% CI); *p*‐value
LVEF, %	57	3.3%, *p* < 0.001	62	−0.3%, *p* = 0.676	2.3 (0.4–4.2); *p* = 0.021
GLS, %	45	−2.4%, *p* < 0.001	47	−1.5%, *p* < 0.001	−0.8 (−1.9 to −0.2); *p* = 0.11
s′, cm/s	57	0.36, *p* = 0.035	62	−0.53, *p* = 0.011	1.12 (1.05–1.20); *p* = 0.001
Stroke volume, ml	57	9.9, *p* < 0.001	61	5.2, *p* = 0.011	6.5 (0.4–12.6); *p* = 0.037

CI, confidence interval; GLS, global longitudinal strain; LVEF, left ventricular ejection fraction; s′, systolic tissue Doppler velocity.

^a^ Muliple linear regression model comparing 12‐month ventricular function parameter by randomized treatment allocation, adjusted for age, sex, modified European Heart Rhythm Association score, history of myocardial infarction and each patient's baseline value for that outcome.

**Figure 2:**
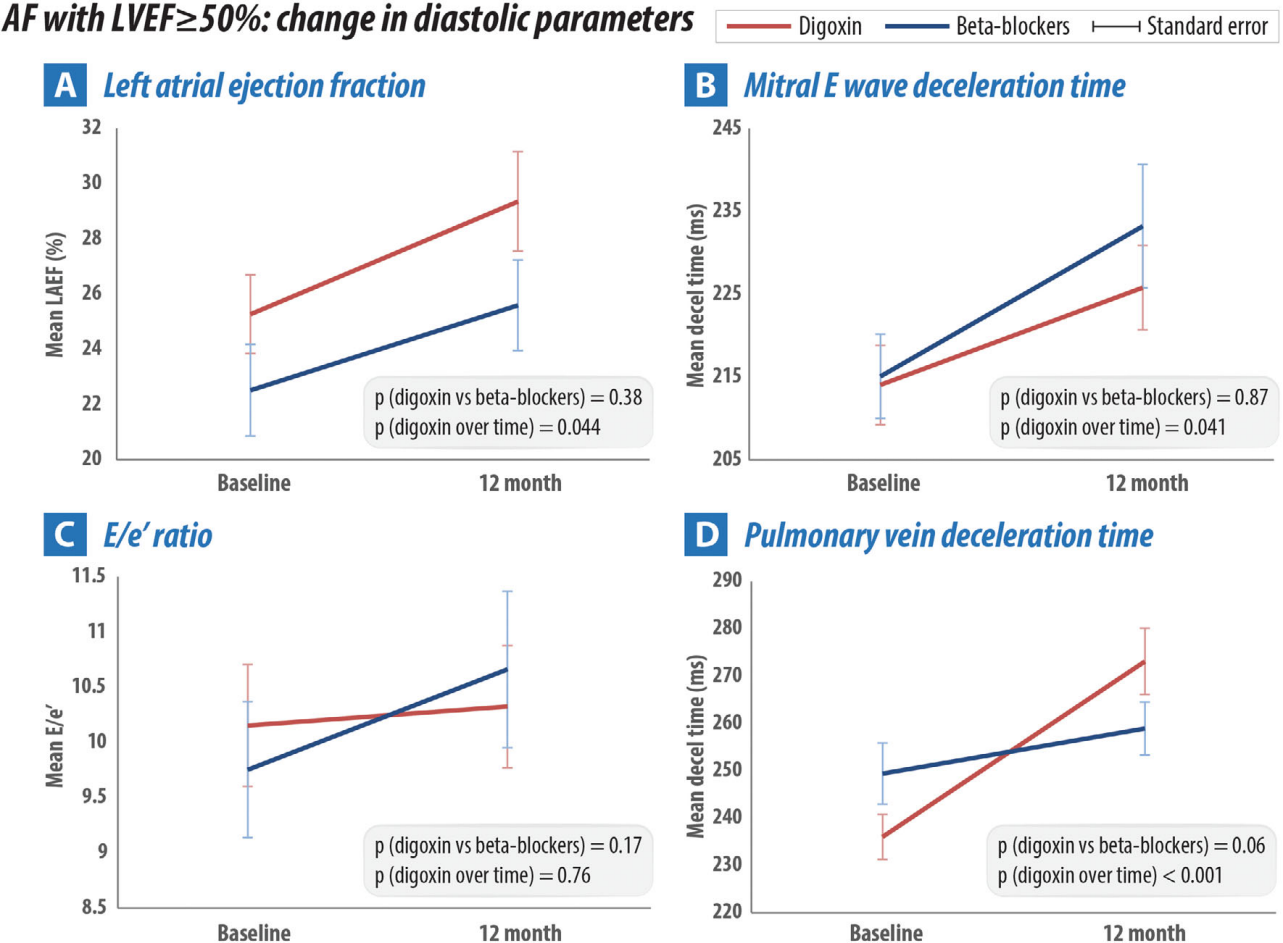
Change in left ventricular diastolic parameters from baseline to 12‐month follow‐up in patients with left ventricular ejection fraction (LVEF) ≥ 50% randomized to beta‐blockers versus digoxin. AF, atrial fibrillation; E/e′, the ratio of mitral E to average of e′ diastolic tissue velocity; LAEF, left atrial ejection fraction.

Significant improvements in diastolic function and LV filling pressure parameters were also noted for patients with baseline LVEF ≥50% randomized to digoxin: from baseline to 12 months an increase in pulmonary vein diastolic deceleration time of 37.1 ms (*p* < 0.001), increase in mitral deceleration time of 11.8 ms (*p* = 0.041) and increase in left atrial ejection fraction of 4.1% (*p* = 0.044). There was no significant change in E/e′ (*p* = 0.76), left atrial reservoir strain (*p* = 0.41), TR V_max_ (*p* = 0.29) or averaged e′ (*p* = 0.32). In patients randomized to beta‐blockers there was an improvement from baseline to 12 months in mitral deceleration time with a mean increase of 18.1 ms (*p* = 0.037). There was no change in left atrial ejection fraction, left atrial reservoir strain or pulmonary vein diastolic deceleration time. There was a worsening of E/e′ (increase of 1.1; *p* = 0.007) and worsening of averaged e′ (decrease by −0.51 cm/s; *p* = 0.038). There was no significant difference in diastolic parameter change between the two treatment arms for patients with preserved LVEF (*Table* *4* and *Figure* *2*).

**Table 4:** Change in left ventricular diastolic function in digoxin versus beta‐blockers with baseline left ventricular ejection fraction ≥50%

Outcome	Digoxin		Beta‐blockers		Digoxin vs. beta‐blockers
	*n*	Mean change from baseline to 12 months, *p*‐value	*n*	Mean change from baseline to 12 months, *p*‐value	Adjusted mean difference^a^ (95% CI); *p*‐value
E/e′	57	1.0, *p* = 0.76	62	1.1, *p* = 0.007	2.72 (0.87–1.02); *p* = 0.17
MV deceleration time, ms	57	11.8, *p* = 0.041	62	18.1, *p* = 0.037	1.01 (0.94–1.08); *p* = 0.87
Average e′, cm/s	57	0.22, *p* = 0.32	62	−0.51, *p* = 0.038	0.57 (−0.21 to 1.36); *p* = 0.15
PV diastolic deceleration time, ms	39	37.1, *p* = <0.001	38	9.5, *p* = 0.26	19.4 (−0.40 to 39.13); *p* = 0.06
LAEF, %	46	4.1%, *p* = 0.044	52	3.1%, *p* = 0.12	2.27 (−2.80 to 7.34); *p* = 0.38
IVRT, ms	54	4.1, *p* = 0.13	55	2.1, *p* = 0.43	0.95 (−4.94 to 6.84); *p* = 0.75
Left atrial reservoir strain, %	45	0.8%, *p* = 0.41	44	1.1%, *p* = 0.17	−0.04 (−2.02 to 1.93); *p* = 0.97
TR V_max_, m/s	18	0.1, *p* = 0.29	33	0.01, *p* = 0.9	0.1 (−0.1 to 0.3); *p* = 0.40

CI, confidence interval; E/e′, the ratio of mitral E to average of e′; e′, diastolic tissue Doppler velocity; IQR, interquartile range; IVRT, isovolumic relaxation time; LAEF, left atrial ejection fraction; MV, mitral valve; PV, pulmonary vein; TR V_max_, maximum tricuspid regurgitation velocity.

^a^ Multiple linear regression model comparing 12‐month ventricular function parameter by randomized treatment allocation, adjusted for age, sex, modified European Heart Rhythm Association score, history of myocardial infarction and each patient's baseline value for that outcome.

### Change in left ventricular function in atrial fibrillation patients with mildly reduced (41–49%) and reduced ejection fraction (≤40%)

For systolic function, there was a significant improvement with digoxin in patients with baseline LVEF 41–49% for LVEF (6.2%; *p* = 0.009) and TDI‐derived s′ (1.25 cm/s; *p* = 0.015). In patients randomized to beta‐blockers, there were significant improvements for those with baseline LVEF 41–49% in LVEF (11.5%; *p* = 0.016) and GLS (−5.63%; *p* = 0.001), and TDI‐derived s′ for baseline LVEF <40% (2.2 cm/s; *p* = 0.045). Across all systolic parameters, the only significant difference between groups was improved TDI‐derived s′ with low‐dose digoxin; adjusted mean difference 1.45 cm/s compared to beta‐blockers (95% CI 1.22–1.73; *p* = 0.002) (online supplementary *Figure* *S3* and *Table* *S4*). There were no differences in diastolic function parameters over time comparing digoxin with beta‐blockers in patients with reduced LVEF (online supplementary *Table* *S5*).

### Interaction of left ventricular ejection fraction with clinical outcomes

**Figure 3:**
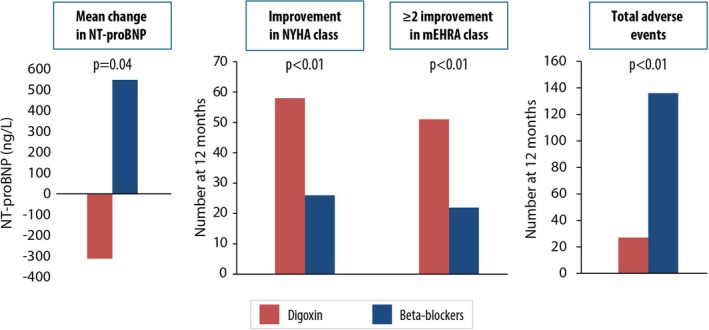
Difference between clinical parameters at 12 months between the beta‐blocker arm and digoxin arm. mEHRA, modified European Heart Rhythm Association; NT‐proBNP, N‐terminal pro‐B‐type natriuretic peptide; NYHA, New York Heart Association.

NT‐proBNP was significantly reduced in patients randomized to digoxin from baseline (median 1091 pg/ml [IQR 710–1522]) to 12‐month follow‐up (960 pg/ml [IQR 626–1531]) but was increased in those randomized to beta‐blockers (1011 pg/ml [IQR 753–1404] to 1250 pg/ml [IQR 847–1890]). Comparing groups, the ratio of geometric means for NT‐proBNP was 0.77 in favour of digoxin (95% CI 0.64–0.92; *p* = 0.004) with no interaction for baseline LVEF (*p*_interaction_ = 0.62) (online supplementary *Table* *S6* and *Figure* *3*). Digoxin led to significant improvement in both NYHA class and mEHRA functional score versus beta‐blockers, with adjusted odds ratios (OR) for a one class or more improvement in NYHA class of 11.32 (95% CI 4.29–29.84; *p* < 0.001) and 4.91 for a 2 or more class improvement in mEHRA score (95% CI 2.36–10.23; *p* < 0.001). In a post‐hoc analysis, more patients in the digoxin group stopped concomitant diuretics, and less started diuretics, compared to those randomized to beta‐blockers (*p* = 0.047), with no difference between groups in the number of participants who changed their diuretic treatment regime (*p* = 0.63) (online supplementary *Table* *S7*). There were no interactions for baseline LVEF (*p*_interaction_ = 0.49 for NYHA class, and = 0.07 for mEHRA score). There were significantly fewer adverse events in patients randomized to digoxin (27 events compared to 136 events in the beta‐blocker arm), with incident rate ratio 0.21 (95% CI 0.13–0.31; *p* < 0.001), and no interactions with baseline LVEF (*p*_interaction_ = 0.13).

## Discussion

This study has demonstrated that patients with AF and a preserved ejection fraction randomized to low‐dose digoxin derive improvements in parameters related to left ventricular function. There was a significantly greater improvement in systolic function in those randomized to digoxin compared to beta‐blockers, where baseline LVEF was preserved (≥50%). Participants of all heart failure phenotypes allocated to digoxin had a significantly greater reduction in NT‐proBNP, better resolution of impaired functional class, and substantially fewer adverse events when compared to beta‐blockers, without any interaction with baseline cardiac function (*Graphical Abstract*). The numbers of patients with mildly reduced or reduced ejection fraction limit the ability to draw conclusions on the effects of digoxin and beta‐blockers on ventricular function.

The number of patients with both AF and heart failure is increasing exponentially, making it important to define effective therapies.^12^, ^13^ However it remains unclear whether treatment strategies should differ between different heart failure phenotypes and the presence or absence of AF.^14^ Recovery or improvement in LVEF has been associated with better prognosis, although studies in patients with AF are limited.^15^

In the TOPCAT trial in which patients with heart failure and LVEF ≥45% (35% with AF) were randomized to spironolactone versus placebo, there was overall a neutral outcome for the primary composite of death from cardiovascular causes, aborted cardiac arrest, or hospitalization. However when stratified by baseline LVEF, there was a benefit demonstrated in patients with an LVEF <50%.^16^ In an individual patient meta‐analysis of RCTs in which patients with heart failure were randomized to beta‐blockers versus placebo, in patients with AF and LVEF ≤49% there was an improvement in LVEF, but across all heart failure phenotypes for patients with AF there was no mortality benefit from beta‐blockers.^8^, ^17^

In the AF with heart failure population from three sodium–glucose co‐transporter 2 (SGLT2) inhibitor trials, there remained a reduction in heart failure hospitalization or cardiovascular death in the SGLT2 inhibitor arm when compared to placebo,^18^ but no significant difference in change in LVEF between the placebo and SGLT2 inhibitor treatment groups, with only one study showing an improvement in LVEF from baseline to follow‐up.^19^

With regards to diastolic function, the IMPRESS‐AF (Improved Exercise Tolerance in Heart Failure With Preserved Ejection Fraction by Spironolactone on Myocardial Fibrosis in Atrial Fibrillation) trial randomized patients with AF and HFpEF to spironolactone versus placebo and found no change in diastolic function measured by E/e′ and no improvement in exercise capacity or quality of life in the spironolactone group.^20^ While we saw no change in E/e′ in response to digoxin, there were significant improvements in other measures of diastolic function including left atrial ejection fraction.

The RATE‐AF trial was launched as the first head‐to‐head randomized trial of beta‐blockers versus digoxin in this patient group. The primary outcome of physical‐related quality of life was no different between digoxin and beta‐blockers, however the majority of secondary outcomes were in favour of digoxin therapy.^9^ This sub‐analysis of the RATE‐AF trial has demonstrated that in patients randomized to low‐dose digoxin there is either an improvement or stabilization in left ventricular systolic function across all heart failure phenotypes, with a greater improvement in systolic function compared to the beta‐blocker arm in patients with LVEF ≥50%. Due to the paucity of data on the validity of left ventricular systolic function for patients in AF^21^ it is unknown whether the improvements seen are clinically relevant. However, this is seen alongside a substantially greater improvement in symptoms of heart failure and AF, greater reduction in NT‐proBNP and significantly fewer treatment‐related adverse events when using digoxin therapy.^22^ The improvements in clinical outcomes were seen in the context of a mean serum digoxin level of 0.78 ng/ml, which falls into a low‐dose regimen. In patients with sinus rhythm and heart failure, higher doses of digoxin are associated with worse prognosis,^23^ although there are no trial data on patients with AF. In a detailed heart rate sub‐study using wearable devices in RATE‐AF participants, there was a similar effect on heart rate from low‐dose digoxin and beta‐blockers, with equivalent effects at rest and with physical activity.^24^

The value of digoxin in patients with heart failure remains controversial due to findings of higher rates of mortality and hospitalization in observational studies. However in RCTs, where prescription bias is addressed, digoxin has a neutral effect on mortality and leads to lower rates of heart failure‐related death and hospitalization.^25^, ^26^ Beta‐blockers also have a neutral effect on mortality, for patients with HFrEF and AF.^17^, ^27^ Previous RCTs assessing the effect of digoxin on long‐term outcomes excluded patients with AF; further studies on the use of cardiac glycosides are due to report, including DECISION^28^ and DIGIT‐HF,^29^ although neither is focussed specifically on patients with concomitant AF. Current guidelines give beta‐blockers and digoxin a similar level of recommendation for long‐term heart rate control, although beta‐blockers are preferred for acute rate control due to their more rapid and predictable heart rate response.^4^

As digoxin inhibits the sodium/potassium‐ATPase pump leading to increases in intracellular calcium, the improvement in systolic function across all heart failure sub‐types with digoxin in this study was perhaps not unexpected. The improvements in diastolic function may be of greater significance in improving symptoms but may not be explained by known pharmacological actions. We cannot exclude effects secondary to rate control, but these effects were not seen in the beta‐blocker‐treated patients. The RATE‐AF population was elderly with multi‐morbid conditions and so may have benefited from the potential senolytic properties of digoxin.^30^

### Study limitations

Although the RATE‐AF trial used an open‐label design, bias was minimized by blinded analysis of all echocardiogram data. Most patients had preserved LVEF at baseline, meaning that conclusions about the effects of digoxin are largely limited to this group. There was no pre‐exclusion criterion for echocardiographic image quality, which meant for analysis of parameters more reliant on image quality not all patients were included. Despite reducing the maximum number of patients for certain analyses, this has made the results more generalizable to the patient population seen in clinical practice. All measurements of systolic and diastolic function were made using an index‐beat which has been shown to reduce variability between measurements compared to conventional averaging.^11^

## Conclusion

Patients with permanent AF and symptoms of heart failure who are treated with low‐dose digoxin benefit from an improvement in left ventricular systolic function across all heart failure phenotypes. In patients with a preserved ejection fraction there was a significantly greater improvement in left ventricular systolic function in patients treated with digoxin, when compared to treatment with beta‐blockers. Digoxin also showed a greater improvement in symptoms related to heart failure and AF with fewer treatment‐related adverse events compared to beta‐blockers, regardless of baseline LVEF.

### Funding

K.V.B. is funded by a British Heart Foundation (BHF) fellowship (FS/CDRF/21/21032). The RATE‐AF trial was funded by the National Institute for Health Research (NIHR) (CDF‐2015‐08‐074). The work is also supported by a BHF Accelerator Award to the University of Birmingham Institute of Cardiovascular Sciences (AA/18/2/34218), the NIHR Birmingham Biomedical Research Centre (NIHR203326), and MRC Health Data Research UK (HDRUK/CFC/01). The opinions expressed in this paper are those of the authors and do not represent any of the listed organizations; none of the organizations had any role in design or conduct of the study (including collection, analysis and interpretation of the data) or any involvement in preparation, review or approval of the manuscript.

**Conflict of interest**: K.V.B. reports receiving funding from the NIHR and the BHF; and receiving personal fees from Health Systems‐Philips UKI. S.K.G. reports funding through the BigData@Heart Innovative Medicines Initiative (grant no.116074). A.J.C. reports receiving consulting fees from Boston Scientific, Abbott, Biosense Webster, Johnson and Johnson and Acesion; and receiving payment for lectures from Menarini, Daiichi Sankyo, Abbott and Boston Scientific. J.N.T. reports payment for manuscript writing from the British Journal of Cardiology. D.K. reports receiving grants from the National Institute for Health Research (NIHR), the British Heart Foundation (BHF), UK National Health Service–Data for R&D‐Subnational Secure Data Environment programme, the European Union‐European Federation of Pharma Industries and Associations Innovative Medicines Initiative BigData@Heart, the European Society of Cardiology, EU Horizon and UKRI, EU/EFPIA Innovative Medicines Initiative and Cook & Wolstenholme Charitable Trust; and receiving personal fees from Bayer, Amomed, and Prothetics Medicine Development. All other authors have nothing to disclose.

## Supporting information


**Appendix S1. Supporting Information**.
