# Supplementary material for: Low‐dose digoxin improves cardiac function in patients with heart failure, preserved ejection fraction and atrial fibrillation – the RATE‐AF randomized trial
Source: Eur J Heart Fail. 2025 Sep 2;27(12):2869–78. doi: 10.1002/ejhf.70022 (PMC12803603; doi:10.1002/ejhf.70022)

# Title: Low-dose digoxin improves cardiac function in patients with heart failure, preserved ejection fraction and atrial fibrillation – the RATE-AF randomised trial

## Supplemental Appendix

Contents

[Title: Low-dose digoxin improves cardiac function in patients with heart failure, preserved ejection fraction and atrial fibrillation – the RATE-AF randomised trial 1](#_Toc204079310)

[Supplemental Appendix 1](#_Toc204079311)

[Supplemental Table 1. Full inclusion and exclusion criteria for the RATE-AF trial 2](#_Toc204079312)

[Supplemental Table 2. Echocardiogram protocol 3](#_Toc204079313)

[Supplemental Table 3. Plain English summary for patients 9](#_Toc204079314)

[Supplemental Table 4. Change in left ventricular systolic and diastolic function in patients with LVEF 41-49% 10](#_Toc204079315)

[Supplemental Table 5. Change in left ventricular systolic and diastolic function in patients with LVEF≤40% 12](#_Toc204079316)

[Supplemental Table 6: Clinical parameters at 12 months and interaction with baseline LVEF 14](#_Toc204079317)

[Supplemental Table 7: Change in diuretic therapy regime from baseline to 12 months according to treatment group 1](#_Toc204079318)

[Supplemental Figure 1. Measurements of left ventricular function performed on an index-beat 2](#_Toc204079319)

[Supplemental Figure 2. Consort diagram 3](#_Toc204079320)

[Supplemental Figure 3. Change in left ventricular systolic parameters from baseline to 12-months in patients with LVEF 41-49% 5](#_Toc204079321)

## Supplemental Table 1. Full inclusion and exclusion criteria for the RATE-AF trial

| Inclusion criteria | Exclusion criteria |
| --- | --- |
| Aged 60 years or over | An established indication for beta-blocker therapy, e.g. myocardial infarction in the last 6 months |
| Permanent AF, characterised (at time of randomisation) as a physician decision for rate-control with no current plans for cardioversion, anti-arrhythmic medication, or ablation therapy | Known contraindications for therapy with beta-blockers or digoxin, e.g. a history of severe bronchospasm that would preclude use of beta-blockers, or known intolerance to these medications |
| Symptoms of breathlessness (New York Heart Association Class II or more) | Baseline heart rate <60 bpm |
| Able to provide written, informed consent | History of second or third-degree heart block |
|  | Supraventricular arrhythmias associated with accessory conducting pathways (e.g. Wolff-Parkinson-White syndrome) or a history of ventricular tachycardia or ventricular fibrillation |
|  | Awaiting pacemaker implantation (including cardiac resynchronisation therapy), have a pacemaker-dependent rhythm or a history of atrioventricular node ablation |
|  | A history of decompensated heart failure (evidenced by need for intravenous inotropes, vasodilators or diuretics) within 14 days prior to randomisation |
|  | A current diagnosis of obstructive hypertrophic cardiomyopathy, myocarditis or constrictive pericarditis |
|  | Undergone heart transplantation, or is on a waiting list for heart transplantation |
|  | Receiving renal replacement therapy (haemodialysis or peritoneal dialysis) |
|  | A history of major surgery, including thoracic or cardiac surgery, within 3 months of randomisation? |
|  | Severe, concomitant non-cardiovascular disease (including malignancy) that is expected to reduce life expectancy |

## Supplemental Table 2. Echocardiogram protocol

| View | Modality | Image acquisition | Number of beats per loop | | Number of repetitions | Sweep speed (mm/s)/ colour scale(cm/s) | Post processing measurement |
| --- | --- | --- | --- | --- | --- | --- | --- |
| PLAX | 2D | LV, AV and MV | 3 | | 1 | n/a | IVSd and LVIDs |
|  | 2D zoom | LVOT and Aortic root | 3 | | 1 | n/a | LVOT diameter and root dimensions |
|  | M-mode | Optimised on-axis LV | 5 | | 6 | 50 mm/s (slow) | LVIDd and LVIDs |
|  |  | Optimised on-axis AoR and LA | 5 | | 6 | 50 mm/s (slow) | LA dimension |
|  | Colour | Colour over MV | 3 | | 1 | 60 cm/s | *quantify any MR |
|  |  | Colour over AV | 3 | | 1 | 60 cm/s | *quantify any AR |
| PLAX RV inflow | 2D | TV and RV inflow | 3 | | 1 | n/a | n/a |
|  | Colour | Colour over TV | 3 | | 1 | 60 cm/s | *quantify any TR |
|  | CW | TV inflow and any regurgitation | n/a | | 1 | 75 mm/s (med) | *TR Vmax |
| PLAX RV outflow | 2D | PV and RV outflow tract | 3 | | 1 | n/a | n/a |
|  | Colour | Colour over PV | 3 | | 1 | 60 cm/s | *quantify any PR |
|  | CW | PV inflow and any regurgitation | n/a | | 1 | 75 mm/s (med) | *quantify any PR or PS |
|  | PW | PV inflow | 3 | | 1 | 75 mm/s(med) | PV Vmax |
| PSAX | 2D | Aortic valve level | 3 | | 1 | n/a | n/a |
|  |  | Basal level | 3 | | 1 | n/a | n/a |
|  |  | Mid-level | 3 | | 1 | n/a | n/a |
|  |  | Apical level | 3 | | 1 | n/a | n/a |
| A4C | 2D | Optimised RV | 3 | | 1 | n/a | RVIDd and RA area |
|  |  | LA | 10 | | 3 | n/a | LA volume, LA transverse dimension and LA longitudinal dimension |
|  |  | Optimised LV | 11 (/6- depending on need for breath hold) | | 3 (/6- depending on need for breath hold) | Adjust speed/res to maximise frame rate | Longitudinal Strain and Simpson’s single plane end-diastolic and end-systolic volume ** |
|  | 2D x-plane | Optimised LV | 6 | | 2 | n/a | Simpson’s biplane end diastolic and end-systolic volume |
|  |  | Optimised LA | 6 | | 2 | n/a | LA biplane volumes |
|  | 3D HVR | Optimised LV | 8 | | 2 | n/a | 3D end-diastolic and end-systolic volumes |
|  | 3D volume | Optimised LV | 4 | | 3 | n/a | 3D end-diastolic and end-systolic volumes |
|  | TDI and PW | Lateral tissue Doppler velocity spectrum | 10 | | 3 | 25 mm/s (min) | s’ and e’ velocities *** |
|  |  | Septal tissue Doppler velocity spectrum | 10 | | 3 | 25 mm/s (min) | s’ and e’ velocities *** |
|  | PW | Mitral valve inflow | 10 | | 3 | 25 mm/s (min) | E and E deceleration time |
|  |  | Pulmonary venous flow | 3 | | 10 | 50 mm/s (slow) | Systolic and diastolic velocity and diastolic deceleration time |
|  | Colour m-mode | Flow through the mitral valve | 5 | | 6 | 50 mm/s (slow)  Colour baseline 40 cm/s | Vp |
|  | M-mode | Motion through RV base | 10 | | 3 | 25 mm/s (min) | TAPSE |
|  | Colour | Colour over Tricuspid valve | 3 | | 1 | 60 cm/s | *quantify regurgitation |
|  |  | Colour over Mitral valve | 3 | | 1 | 60 cm/s | *quantify regurgitation |
| A5C | Colour | Colour over aortic valve | 3 | | 1 | 60 cm/s | *quantify regurgitation |
|  | CW | Aortic valve flow | n/a | | 1 | 75 mm/s (med) | AV Vmax ** |
|  | PW | LVOT flow | 5 | | 6 | 50 mm/s (slow) | LVOT Vmax and LVOT VTI *** |
|  |  | LVOT and MV flow | 3 | | 10 | 75 mm/s (med) | IVRT |
| A2C | 2D | Optimised LV | 11 / (6- depending on need for breath hold) | | 3 / (6- depending on need for breath hold) | n/a | Simpson’s single plane end-diastolic and end systolic volume and  Longitudinal strain |
| A3C | 2D | Optimised LV | 11 / (6- depending on need for breath hold) | | 3 / (6- depending on need for breath hold) | n/a | Longitudinal strain |
| Subcostal 4C | 2D | 4 chambers of the heart | 3 | | 1 | n/a | n/a |
| Subcostal SAX | 2D | IVC | 3 | | 1 | n/a | IVC diameter |
|  |  | IVC collapse with respiration | Suprasternal | | 1 | 3 | IVC collapse with respiration |
| Suprasternal | 2D | Arch | 3 | | 1 | n/a | Arch dimensions |
|  | Colour | Colour over Desc Ao | 3 | | 1 | 60 cm/s | n/a |
|  | CW | Flow down Desc Ao | n/a | | 1 | 75 mm/s (med) | n/a |
|  | PW | Flow at prox Desc Ao | n/a | | 1 | 75 mm/s (med) | n/a |
| **Patient sits up and operator gets up and leaves the room before returning to do the measurements to test intra-operator variability. All following loops should be labelled “IOV1”** | | | | | | | |
| A4C | 2D | Optimised LV | 11/ (6- depending on need for breath hold) | 1 / (2- depending on need for breath hold) | | n/a | Longitudinal strain ** |
|  | TDI and PW | Lateral tissue velocity spectrum | 10 | 1 | | 25 mm/s (min) | s’ and e’ velocities *** |
|  |  | Septal tissue velocity spectrum | 10 | 1 | | 25 mm/s (min) | s’ and e’ velocities *** |
|  | PW | Mitral valve inflow | 11 | 1 | | 25 mm/s (min) | E and E deceleration time |
| A2C | 2D | Optimised LV | 11/ (6- depending on need for breath hold) | 1 / (2- depending on need for breath hold) | | n/a | Longitudinal strain |
| A3C | 2D | Optimised LV | 11 / (6- depending on need for breath hold) | 1 / (2- depending on need for breath hold) | | n/a | Longitudinal strain |
| **On every 8 patients a second operator should take the following acquisitions and all loops should be labelled “IOV2”** | | | | | | | |
| A4C | 2D | Optimised LV | 11 / (6- depending on need for breath hold | 1 (2- depending on need for breath hold) | | n/a | Longitudinal strain |
|  | TDI and PW | Lateral tissue velocity spectrum | 10 | 1 | | 25 mm/s (min) | s’ and e’ velocities *** |
|  |  | Septal tissue velocity spectrum | 10 | 1 | | 25 mm/s (min) | s’ and e’ velocities *** |
|  | PW | Mitral valve inflow | 10 | 1 | | 25 mm/s (min) | E and E deceleration time |
| A2C | 2D | Optimised LV | 11 / (6- depending on need for breath hold | 1 (2- depending on need for breath hold) | | n/a | Longitudinal strain |
| A3C | 2D | Optimised LV | 11 / (6- depending on need for breath hold | 1 (2- depending on need for breath hold) | | n/a | Longitudinal strain |

Abbreviations:, 3D= three dimensional; A4C= Apical 4 Chamber; A2C= Apical 2 Chamber, A3C= Apical 4 Chamber; AoR= Aortic root; AR= aortic regurgitation; AV= Aortic Valve; CW= continuous wave; Desc Ao= descending aorta; IVC= inferior vena cava; IVRT= isovolumic relaxation time; IVSd= Inter-ventricular septum diameter, LA= Left atrium; LVIDd= left ventricular internal diameter in diastole; LVIDs= left ventricular internal diameter in systole; LV= left ventricle; LVOT= Left Ventricular Outflow Tract; LVPWd= Left Ventricular Posterior Wall diameter; MR=mitral regurgitation, MV=mitral valve, MR= mitral regurgitation; RV= right ventricle, PLAX= Parasternal Long Axis; PR=

## Supplemental Table 3. Plain English summary for patients

| What is atrial fibrillation (AF) and heart failure? |
| --- |
| AF is a common heart condition that leads to an irregular and often rapid heart rate. AF can reduce the function of the heart resulting in patients developing a condition called heart failure. Heart failure occurs when either, the heart doesn’t pump or relax as well as it should. This results in the body not receiving enough oxygen, causing patients to develop symptoms such as being out of breath, dizziness and swelling in their feet and ankles. |
| What was the purpose of this study? |
| The aim of the RATE-AF trial was to compare two treatments used to control heart rate, called beta-blockers and digoxin. In this study we wanted to see if there was a difference in how the treatments affected heart function. Before the patients started their treatment and after 12 months of treatment, an ultrasound scan of the heart called an echocardiogram, was performed to measure how well the heart pumped and relaxed. |
| What did we find out? |
| Digoxin improved heart pump function in all patients with AF and heart failure. In patients with AF and heart failure caused by a heart which is unable to relax properly, the treatment digoxin improved the pump function of the heart more than beta-blockers. Digoxin was also found to cause fewer side effects than beta-blockers and have a greater improvement on symptoms related to AF and heart failure. |
| Patient support |
| Patient information from the British Heart Foundation: <https://www.bhf.org.uk/heart-health/conditions/atrial-fibrillation>  Education and support groups for patients and carers from the Heart Rhythm Alliance: <https://www.heartrhythmalliance.org/> |

## Supplemental Table 4. Change in left ventricular systolic and diastolic function in patients with LVEF 41-49%

| **Outcome** | **Digoxin** | | **Beta-blockers** | | **Digoxin vs beta-blockers** | |
| --- | --- | --- | --- | --- | --- | --- |
|  | **N** | **Mean change from baseline to 12-months, p-value** | **N** | **Mean change from baseline to 12-months, p-value** | **Adjusted mean difference* (95% CI)** | **P-value*** |
| **LVEF (%)** | 11 | 6.2%, p=0.009 | 4 | 11.5%, p=0.016 | -4.0 (-15.0 to 7.0) | 0.41 |
| **GLS (%)** | 10 | -0.20%, p=0.81 | 3 | -5.63%, p=0.001 | 5.54 (-1.13 to -12.2) | 0.08 |
| **S’ (cm/s)** | 11 | 1.25 cm/s, p=0.015 | 4 | -2.60 cm/s, p=0.12 | 1.45 (1.22 to 1.73) | 0.002 |
| **Stroke volume (ml)** | 11 | 6.45 mls, p=0.22 | 4 | 4.67 mls, p=0.43 | 9.17 (-14.24 to 32.58) | 0.36 |
| **E/e’** | 11 | 1.00, p=0.61 | 4 | 2.28, p=0.043 | 0.87 (0.51 to 1.48) | 0.55 |
| **MV deceleration time (ms)** | 11 | 19.8 ms, p=0.15 | 4 | 23.0 ms, p=0.17 | 1.08 (0.81 to 1.43) | 0.54 |
| **Average e’ (cm/s)** | 11 | 0.92 cm/s, p=0.21 | 4 | -1.6 cm/s, p=0.051 | 0.96 (-3.63 to 5.56) | 0.63 |
| **Pulmonary vein diastolic deceleration time (ms)** | 7 | -9.8 ms, p=0.55 | 3 | 43.8 ms, p= 0.20 | 80.1 (-1009 to 1169) | 0.52 |
| **LAEF (%)** | 10 | 7.4 %, p=0.13 | 4 | -1.1%, p=0.85 | -0.62 (-20.95 to 19.72) | 0.94 |
| **IVRT (ms)** | 11 | 0.96 ms, p=0.91 | 3 | -0.67 ms, p=0.94 | 11.2 (-31.94 to 54.26) | 0.54 |
| **Left atrial reservoir strain (%)** | 9 | 3.3%, p=0.10 | 1 | 1.7%, p=. | -0.67 (-51.2 to 49.9) | 0.96 |

*Multiple linear regression model comparing 12-month ventricular function parameter by randomised treatment allocation, adjusted for age, sex, modified EHRA score, history of myocardial infarction and each patient’s baseline value for that outcome. Abbreviations: GLS= global longitudinal strain; IQR= interquartile range; LVEF= left ventricular ejection fraction; N= number of patients at 12 months; s’= systolic Tissue Doppler velocity; E/e’= the ratio of mitral E to average of e’; e’= diastolic tissue velocity; IVRT= isovolumic relaxation time; LAEF= left atrial ejection fraction; MV= mitral valve; PV= pulmonary vein;

## Supplemental Table 5. Change in left ventricular systolic and diastolic function in patients with LVEF≤40%

| **Outcome** | **Digoxin** | | **Bisoprolol** | | **Digoxin vs beta-blockers** | |
| --- | --- | --- | --- | --- | --- | --- |
|  | **N** | **Mean change from baseline to 12-months, p-value** | **N** | **Mean change from baseline to 12-months, p-value** | **Adjusted mean difference* (95% CI)** | **P-value*** |
| **LVEF (%)** | 5 | 4.5%, p=0.33 | 6 | 16.5%, p= 0.06 | 13.1% (-25.0 to 51.1) | 0.35 |
| **GLS (%)** | 4 | -2.00%, p=0.14 | 5 | -3.74%, p=0.18 | 0.39% (-13.52 to 14.29) | 0.78 |
| **S’ (cm/s)** | 5 | -0.27 cm/s, p=0.67 | 6 | 2.19 cm/s, p=0.05 | 1.07 cm/s (0.44 to 2.63) | 0.82 |
| **Stroke volume (ml)** | 5 | 4.60ml, p=0.34 | 6 | 20.7ml, p=0.07 | -7.3 ml (-58.7 to 44.1) | 0.68 |
| **E/e’** | 5 | 0.56, p=0.69 | 6 | -2.68, p=0.20 | 0.33 (0.06 to 1.95) | 0.14 |
| **MV deceleration time (ms)** | 5 | 8.8ms, p=0.52 | 6 | 56.8ms, p=0.06 | 1.21 ms (0.69 to 2.11) | 0.37 |
| **Average e’ (cm/s)** | 5 | 0.2cm/s, p=0.79 | 6 | 1.1 cm/s, p=0.46 | 9.27 (-7.33 to 25.87) | 0.17 |
| **Pulmonary vein diastolic deceleration time (ms)** | 2 | 36.7 ms, p=0.58 | 4 | 21.7 ms, p=0.30 | - | - |
| **LAEF (%)** | 5 | 15.5%, p=0.005 | 6 | 9.3%, p=0.22 | 9.8 (-59.3 to 78.9) | 0.68 |
| **IVRT (ms)** | 5 | 8.6 ms, p= 0.52 | 6 | 1.0 ms, p= 0.92 | -30.9 ms (-210.7 to 148.9) | 0.62 |
| **Left atrial reservoir strain (%)** | 4 | 1.8%, p=0.59 | 6 | 8.6%, p=0.19 | 10.1% (-41.1 to 61.4) | 0.48 |

*Multiple linear regression model comparing 12-month ventricular function parameter by randomised treatment allocation, adjusted for age, sex, modified EHRA score, history of myocardial infarction and each patient’s baseline value for that outcome. Abbreviations: GLS= global longitudinal strain; IQR= interquartile range; LVEF= left ventricular ejection fraction; N= number of patients at 12 months; s’= systolic Tissue Doppler velocity; E/e’= the ratio of mitral E to average of e’; e’= diastolic tissue velocity; IVRT= isovolumic relaxation time; LAEF= left atrial ejection fraction; MV= mitral valve; PV= pulmonary vein;

## Supplemental Table 6: Clinical parameters at 12 months and interaction with baseline LVEF

| **Parameter** | **Statistic** | **Digoxin** | **Beta-blocker** | **Digoxin vs beta-blockers**  **Adjusted mean difference at (AMD), odds ratio (OR) or incidence risk ratio (IRR), with 95% CI** | **Interaction p-value for baseline LVEF** |
| --- | --- | --- | --- | --- | --- |
| **NT-proBNP** | Mean change (SD) | -310  (1781) | 551  (2622) | *AMD 0.77 (0.64 to 0.92); p=0.004 | p=0.62 |
| **Improvement in NYHA class** | n (%) | 58 (79%) | 26 (36%) | † OR 11.32 (4.29 to 29.84); p<0.001 | p=0.49 |
| **Two class or more improvement in mEHRA score** | n (%) | 51 (70%) | 22 (31%) | † OR 4.91 (2.36 to 10.23); P<0.001 | p=0.07 |
| **Total number of adverse events** | n | 27 | 136 | ‡ IRR 0.21 (0.13 to 0.31); p<0.001 | p=0.13 |

*Multiple linear regression model comparing 12-month clinical parameter by randomised treatment allocation, adjusted for age, sex, modified EHRA score, history of myocardial infarction, baseline LVEF and each patient’s baseline value for the outcome of interest. A sensitivity analysis controlling for baseline creatinine and BMI in addition to the above co-variables was performed: AMD 0.78 (95% CI 0.65 to 0.93; p= 0.007); with no interaction between BMI (p=0.87) and creatinine (interaction p=0.72) with the randomised allocation group.

† Poisson regression model comparing 12-month clinical parameter by randomised treatment allocation, adjusted for age, sex, modified EHRA score, history of myocardial infarction, baseline LVEF and each patient’s baseline value for the outcome of interest.

‡ Incidence risk ratio model comparing 12-month total adverse events over time by randomised treatment allocation, adjusted for age, sex, modified EHRA score, history of myocardial infarction and baseline LVEF.

## Supplemental Table 7: Change in diuretic therapy regime from baseline to 12 months according to treatment group

| **Treatment allocation** | **Diuretic regime from baseline to 12 months** | | | **Difference between groups for participants who started diuretic therapy** |
| --- | --- | --- | --- | --- |
|  | **Stopped, n (%)** | **No change, n (%)** | **Started, n (%)** | **Pearson chi2** |
| **Digoxin** | 5 (5%) | 35 (71%) | 9 (18%) | 6.1, p=0.047 |
| **Beta-blocker** | 0 (0%) | 38 (73%) | 14 (27%) |  |

This is from a post-hoc analysis

## Supplemental Figure 1. Measurements of left ventricular function performed on an index-beat


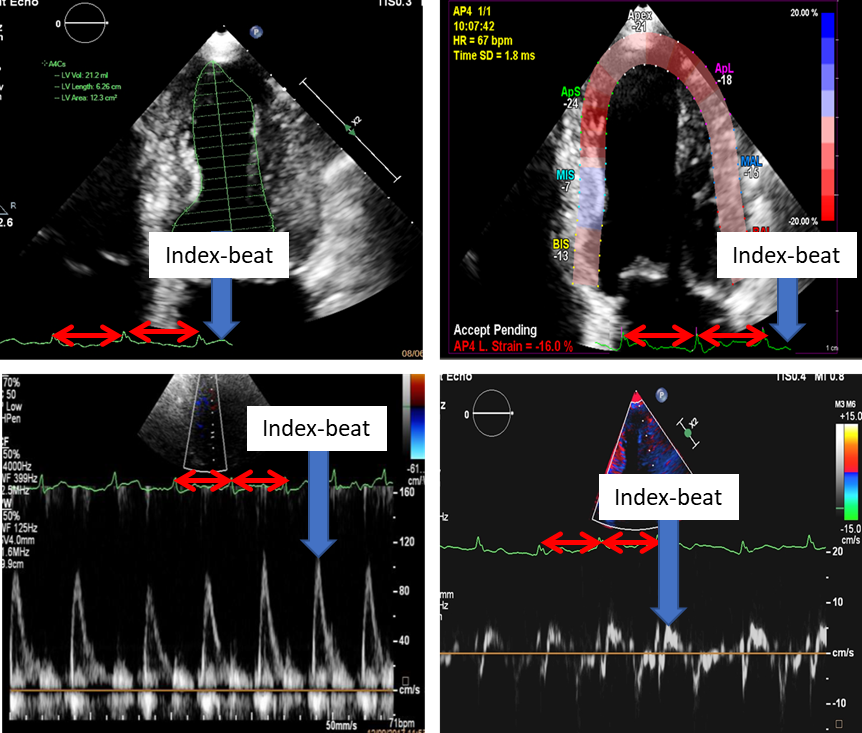


## Supplemental Figure 2. Consort diagram


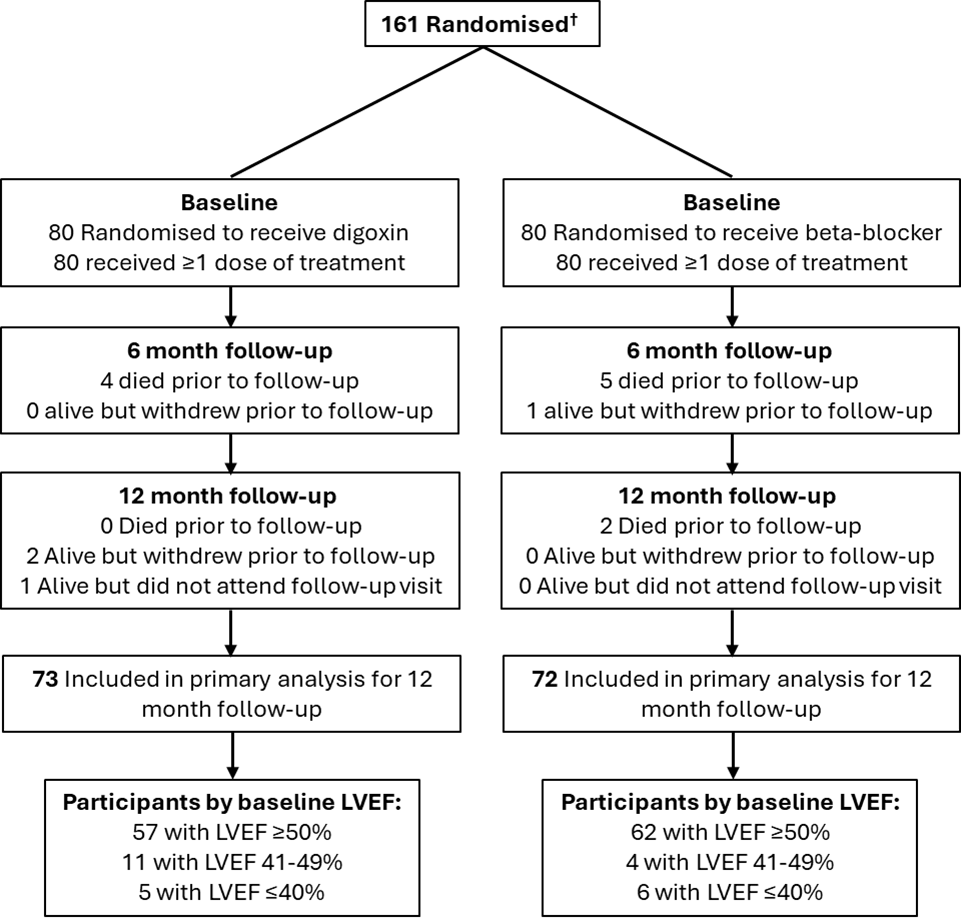


†Randomization included minimization to balance sex and modified European Heart Rhythm Association class at baseline. One person withdrew after randomization before receiving any therapy.

Abbreviations: LVEF= left ventricular ejection fraction

Supplemental Figure 3. Change in left ventricular systolic parameters from baseline to 12-months in patients with LVEF 41-49%
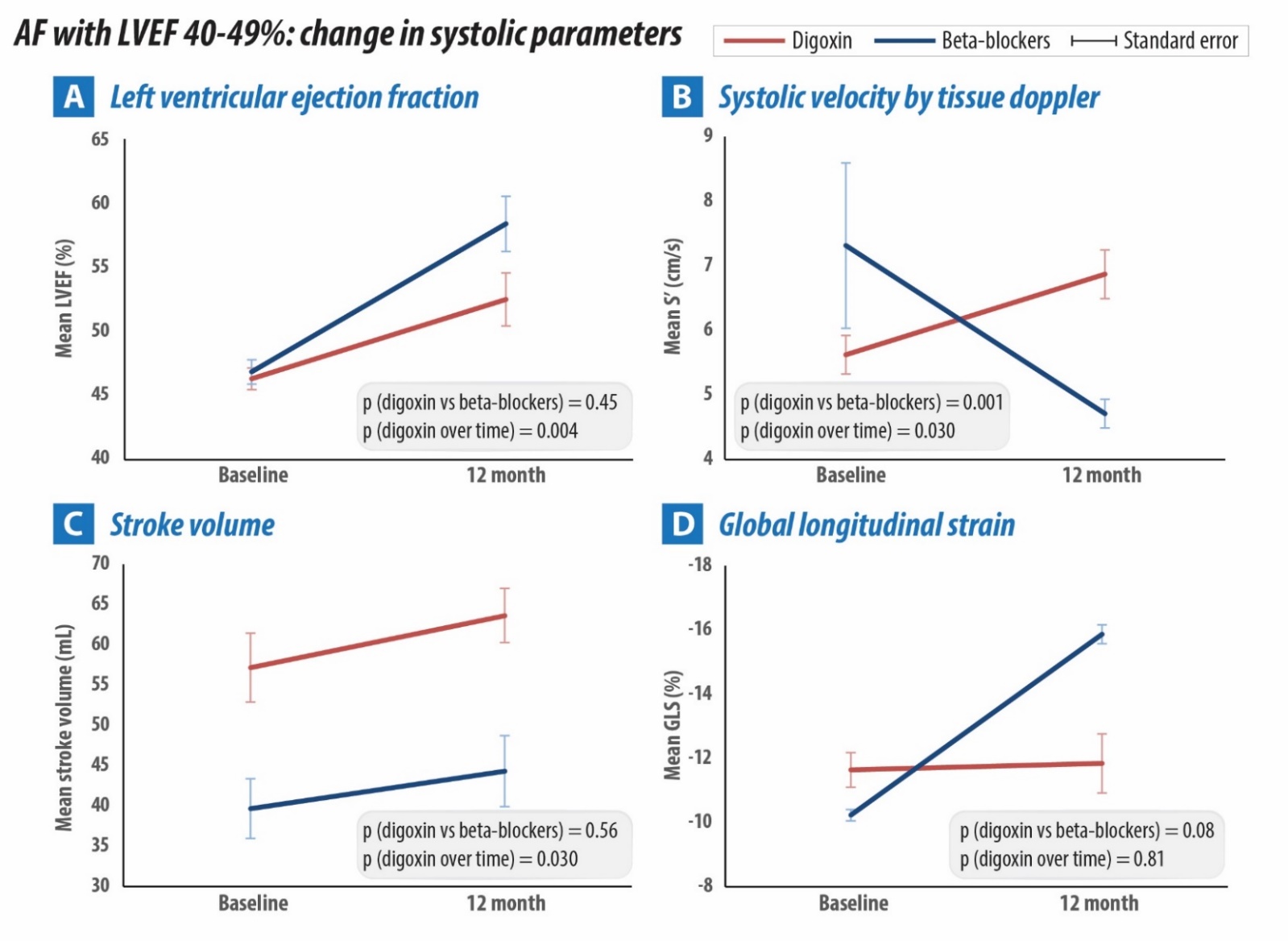

Supplement: Supplementary file 1 — Appendix S1. Supporting Information. [file EJHF-27-2869-s001.docx]
